# Epidemiological Indicators of Tuberculosis in the Federal District, Brazil: Treatment Follow-Up and Case Closure Outcomes From 2015 to 2022

**DOI:** 10.7759/cureus.101922

**Published:** 2026-01-20

**Authors:** Débora de Freitas Britto Rêgo

**Affiliations:** 1 Public Health, Universidade Católica de Brasília, Brasília, BRA

**Keywords:** brazil, covid-19 pandemic, epidemiology, health services access, public health surveillance, pulmonary tuberculosis, tuberculosis

## Abstract

Introduction

Tuberculosis (TB) remains a relevant public health problem in Brazil, and in the Federal District it continues to pose challenges related to diagnosis, treatment adherence, and continuity of care. Monitoring epidemiological indicators is essential to identify weaknesses in TB control and to guide health system responses.

Objective

To analyze the epidemiological situation of TB in the Federal District, Brazil, from 2015 to 2022, focusing on patient profile, treatment monitoring, and case closure outcomes, as well as to assess the impact of the COVID-19 pandemic on TB care.

Methods

A descriptive epidemiological study was conducted using secondary data from the National Notifiable Diseases Information System, accessed through the Brazilian Ministry of Health database. The variables analyzed included the annual incidence of new TB cases, sociodemographic characteristics, type of case entry, directly observed treatment (DOT) coverage, and treatment outcomes. Population estimates were based on official census data.

Results

A marked reduction in TB case detection and treatment success indicators was observed from 2020 onward, coinciding with the COVID-19 pandemic. Decreased cure rates, increased treatment abandonment, reduced coverage of DOT, and higher re-entry after abandonment were identified in the Federal District. Although a partial recovery in incidence occurred in 2022, the indicators did not return to pre-pandemic levels.

Conclusion

The findings demonstrate the negative impact of the COVID-19 pandemic on TB surveillance and care in the Federal District, highlighting the need to strengthen monitoring strategies, improve treatment adherence, and reorganize health services to mitigate long-term effects on TB control.

## Introduction

Epidemiological surveillance comprises a set of actions aimed at generating knowledge, detecting changes in health determinants, and guiding disease prevention and control measures. In Brazil, tuberculosis (TB) is a compulsory notifiable disease, and information related to its occurrence, monitoring, and outcomes is systematically recorded in the National Notifiable Diseases Information System (SINAN), which plays a central role in supporting surveillance and public health decision-making [1].

TB is an infectious disease caused by *Mycobacterium tuberculosis*, transmitted mainly through airborne particles expelled by individuals with active pulmonary or laryngeal disease. Transmission persists while bacilli are present in respiratory secretions but is substantially reduced after the initiation of appropriate treatment, highlighting the importance of early diagnosis and continuous follow-up to achieve cure and interrupt transmission chains [2].

Despite advances in diagnosis and treatment, TB remains strongly associated with social determinants of health, disproportionately affecting vulnerable populations and reflecting social inequalities [3]. In response to the persistent global burden of TB, the WHO launched the End TB Strategy in 2015, establishing long-term targets to reduce TB incidence by 90% and mortality by 95% by 2035, compared with 2015 levels [4]. These goals were designed to be achieved progressively over several decades, providing a global framework for monitoring trends and guiding national tuberculosis control efforts. Brazil is among the countries with a high TB burden and, in alignment with global recommendations, implemented the National Plan to End Tuberculosis as a Public Health Problem, which defines national strategies and targets adapted to the Brazilian context [5].

Before the COVID-19 pandemic, Brazil showed gradual improvements in TB control indicators [6]; however, the pandemic significantly disrupted health services worldwide. Reduced access to health care, reallocation of health resources, and decreased health-seeking behavior compromised TB diagnosis, notification, treatment adherence, and follow-up [7]. Evidence indicates that the observed reduction in TB case detection during this period reflects underdiagnosis rather than a true reduction in disease burden [8].

Understanding the epidemiological impact of these disruptions is essential to guide post-pandemic recovery strategies. In this context, the present study aimed to analyze descriptive, population-based epidemiological indicators of TB in the Federal District, Brazil, from 2015 to 2022, focusing on patient profile, treatment monitoring, and treatment outcomes, and comparing local trends with national patterns.

## Materials and methods

Study design 

This is a descriptive, ecological, population-based study based on the analysis of aggregated secondary data obtained from official health information systems. The study followed a retrospective design and aimed to describe the epidemiological profile, incidence, and treatment outcomes of TB cases in the Federal District, Brazil, over an eight-year period.

Data source 

Data were obtained from the Brazilian National Notifiable Diseases Information System (Sistema de Informação de Agravos de Notificação (SINAN)), accessed through the Department of Informatics of the Unified Health System (DATASUS). The database includes information on all notified and confirmed TB cases in Brazil, according to the year of diagnosis. The study period comprised data from January 2015 to December 2022.

Study population 

The study population consisted of confirmed TB cases reported to SINAN among individuals aged 20 years or older who were residents of the Federal District, Brazil, during the study period. This age cutoff was adopted due to the predefined age group structure of the DATASUS platform, in which the first age category that includes exclusively adults corresponds to individuals aged 20-29 years, while the preceding category (15-19 years) includes minors. Only cases officially registered and validated in the SINAN database were considered.

Inclusion and exclusion criteria 

Inclusion criteria comprised confirmed TB cases registered in SINAN with complete records, referring to residents of the Federal District between 2015 and 2022. Exclusion criteria included incomplete or inconsistent records, notifications outside the study period, and cases involving individuals younger than 20 years of age.

Variables analyzed 

The variables analyzed in this study included the annual incidence of new TB cases, calculated per 100,000 inhabitants. Sociodemographic characteristics were assessed based on sex and age group. The type of case entry into the surveillance system was evaluated and classified as new case, relapse, re-entry after treatment abandonment, or transfer. The proportion of patients undergoing directly observed treatment (DOT) was analyzed as an indicator of treatment follow-up. Treatment outcomes were examined and categorized as cure, treatment abandonment or treatment failure, death due to TB, death from other causes, transferred or not evaluated, and drug-resistant TB or change of therapeutic regimen.

Population estimates

Population estimates used to calculate TB incidence rates were obtained from official demographic data published by the Brazilian Institute of Geography and Statistics (Instituto Brasileiro de Geografia e Estatística (IBGE)), based on the 2010 and 2022 Brazilian Demographic Censuses. According to these censuses, the population of the Federal District increased from 2,570,160 inhabitants in 2010 to 2,817,381 inhabitants in 2022.

To estimate the annual population size for each year of the study period (2015-2022), an exponential population growth model was applied. This approach is widely recognized in epidemiological studies for intercensal estimation when only two census points are available, as it assumes a continuous and constant rate of change. Although potential pandemic-related fluctuations in internal migration and mortality patterns are noted, the exponential model provides a standardized and mathematically robust denominator for calculating incidence trends, maintaining methodological consistency with Brazilian national health surveillance standards. The average annual population growth rate of 0.768% was calculated based on the population variation between the two census years and was applied uniformly across the study period.

These annual population estimates were used as denominators for the calculation of TB incidence coefficients, expressed per 100,000 inhabitants.

Data analysis 

Data were extracted from the DATASUS platform and subsequently organized and cleaned to ensure consistency and completeness. Descriptive data analyses were performed to characterize the study population and TB cases over the study period. Absolute frequencies and proportions were calculated for categorical variables, while incidence coefficients were calculated for each year by dividing the number of new TB cases by the estimated population and multiplying the result by 100,000 inhabitants.

Temporal trends in TB incidence and treatment outcomes were described using year-by-year comparisons. Results were summarized and presented in tables and figures to facilitate interpretation. No inferential statistical tests were performed, as the objective of the study was descriptive in nature.

Ethical considerations 

This study was conducted using publicly available, de-identified secondary data obtained from official national health information systems. According to Brazilian regulations, research based exclusively on public-domain secondary data does not require individual informed consent. All ethical principles related to data confidentiality, integrity, and responsible use of information were strictly observed throughout the research process.

## Results

Incidence of TB in the Federal District

**Figure 1:**
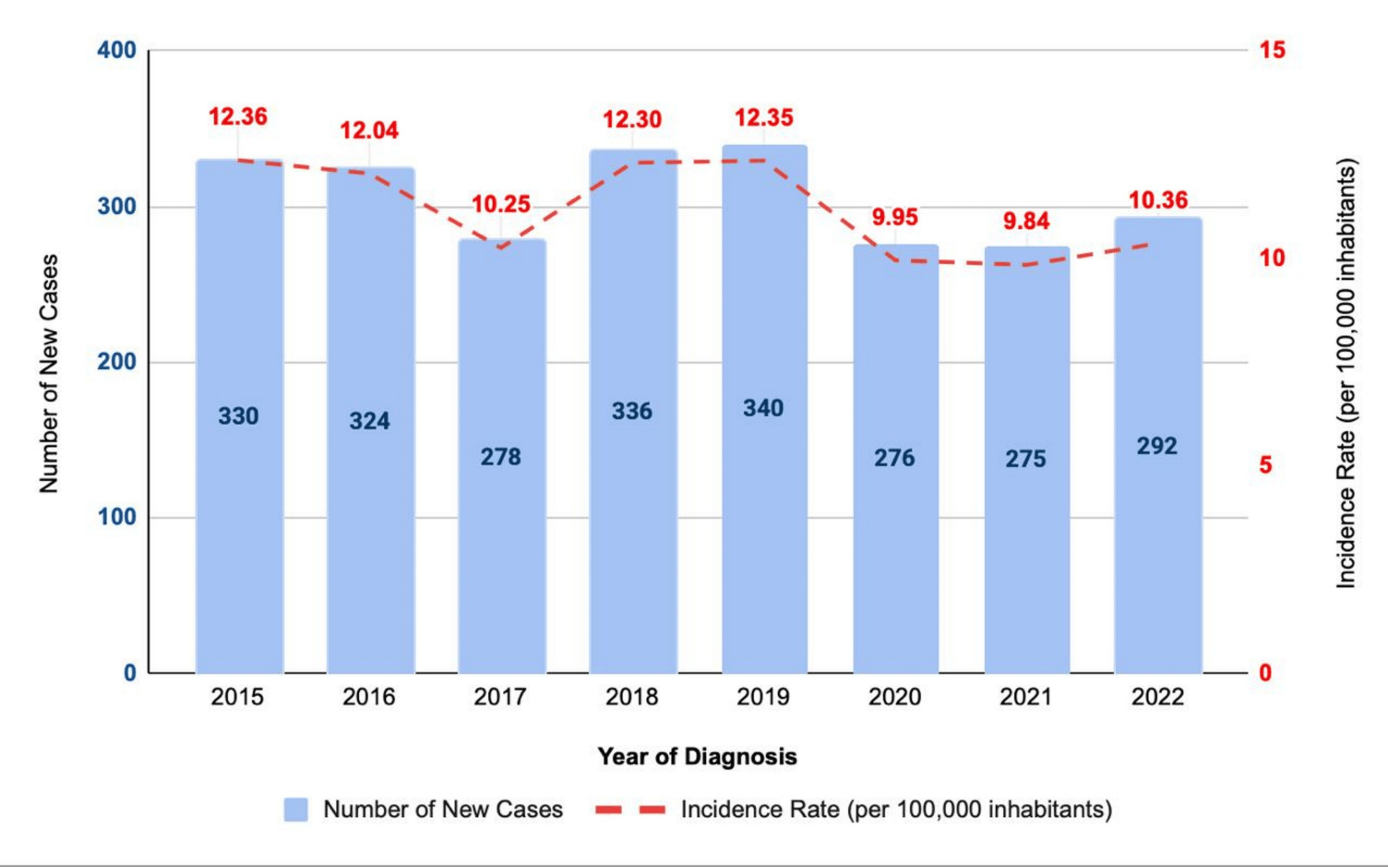
Annual incidence of new tuberculosis cases in the Federal District, Brazil, from 2015 to 2022. The figure illustrates the temporal trend in newly reported tuberculosis cases, highlighting the reduction observed during the COVID-19 pandemic and the partial recovery in 2022.

The first aspect evaluated was the number of new TB cases and the corresponding incidence coefficient. In the Federal District (Figure 1), both the number of newly reported cases and TB incidence rates remained relatively stable between 2015 and 2019, with only minor annual fluctuations and no major changes in the overall trend. An exception was observed in 2017, when a reduction in the number of new cases was noted compared to the surrounding years.

In 2020, a noticeable decline in both the number of newly reported cases and the incidence of TB was observed compared to the two preceding years. This downward trend persisted throughout 2021, indicating a sustained reduction in case detection during this period. In 2022, the first signs of recovery were identified, with an increase in the number of new cases and incidence rates compared to 2020 and 2021; however, these values did not reach the incidence levels observed in the pre-pandemic period.

Sociodemographic characteristics

**Figure 2:**
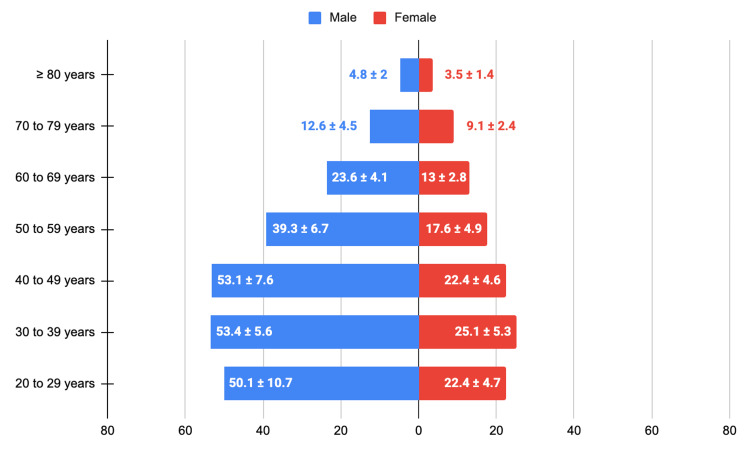
Mean (± SD) number of new tuberculosis cases by age group and sex, Federal District, Brazil, 2015-2022. Values represent the arithmetic mean and SD of the annual number of newly reported tuberculosis cases, stratified by age group and sex, over the eight-year study period (2015-2022).

Throughout the study period (2015-2022), TB predominantly affected male individuals. A total of 2,976 TB cases were reported, of which 2,048 (68.8%) occurred among males and 928 (31.2%) among females, resulting in an overall male-to-female ratio of approximately 2.2:1. This pattern was consistent across all age groups analyzed (Figure 2), indicating a sustained predominance of TB among males over time.

Regarding age distribution, TB cases were concentrated mainly among adults of working age. Individuals aged 20 to 49 years represented the largest proportion of cases throughout the study period, accounting for an average of approximately 67% of all reported cases in the Federal District. Within this group, a relatively homogeneous distribution was observed across the age subgroups of 20-29, 30-39, and 40-49 years. Adults aged 50 to 59 years accounted for an additional 15.4% of cases. A marked reduction in the number of cases was observed among individuals aged 70 years or older. No substantial changes in the overall age distribution pattern were observed over time, including during the COVID-19 pandemic years. It should be noted that the exclusion of individuals under 20 years of age, due to the predefined age group structure of the data source, may partially influence the observed age distribution in this analysis.

To calculate annual TB incidence rates, population estimates for the Federal District were derived from official demographic data. According to the Brazilian Demographic Census, the population of the Federal District was 2,570,160 inhabitants in 2010 and increased to 2,817,381 inhabitants in 2022. Based on these census data points, an exponential population growth model was applied to estimate the annual population size for each year of the study period. The average annual population growth rate applied for the Federal District was 0.768%.

Using this approach, annual population estimates were generated for the years 2015 to 2022 and served as denominators for the calculation of TB incidence coefficients, expressed per 100,000 inhabitants. These demographic estimates allowed for a standardized assessment of TB incidence over time, accounting for population growth during the study period.

Type of case entry

After grouping the total number of TB cases diagnosed each year, cases were classified according to the type of entry (Table 1), including new cases, relapse, re-entry after treatment abandonment, transfer, post-mortem diagnosis, and unknown classification. In the Federal District, new cases accounted for the majority of TB notifications throughout the study period.

**Table 1:** Confirmed tuberculosis cases by type of case entry according to year of diagnosis, Federal District, Brazil, 2015-2022. This table presents the distribution of tuberculosis cases according to the type of entry recorded in the SINAN system, by year of diagnosis, in the Federal District from 2015 to 2022. Values are presented as absolute numbers (n) and percentages (%), calculated in relation to the total number of cases for each year. Values are presented as absolute numbers (n) and percentages (%).

Year of diagnosis	New case, n (%)	Relapse, n (%)	Re-entry after loss to follow-up, n (%)	Unknown, n (%)	Transfer, n (%)	Post-mortem, n (%)	Total, n (%)
2015	330 (89.2)	6 (1.6)	19 (5.1)	0 (0.0)	15 (4.1)	0 (0.0)	370 (100)
2016	324 (88.0)	15 (4.1)	17 (4.6)	1 (0.3)	11 (3.0)	0 (0.0)	368 (100)
2017	278 (81.8)	19 (5.6)	19 (5.6)	9 (2.6)	12 (3.5)	3 (0.9)	340 (100)
2018	336 (84.0)	25 (6.3)	23 (5.8)	5 (1.3)	7 (1.8)	4 (1.0)	400 (100)
2019	340 (84.2)	13 (3.2)	30 (7.4)	8 (2.0)	11 (2.7)	2 (0.5)	404 (100)
2020	276 (79.1)	17 (4.9)	26 (7.4)	7 (2.0)	19 (5.4)	4 (1.1)	349 (100)
2021	275 (78.8)	13 (3.7)	35 (10.0)	8 (2.3)	18 (5.2)	0 (0.0)	349 (100)
2022	292 (73.7)	12 (3.0)	53 (13.4)	9 (2.3)	27 (6.8)	3 (0.8)	396 (100)

Between 2015 and 2019, new cases represented, on average, 85.4% of all reported TB cases. From 2020 onward, a reduction in the proportion of new cases was observed. In 2020, new cases accounted for 79.08% of notifications, decreasing to 78.80% in 2021 and 73.74% in 2022.

Relapse cases remained relatively stable over the years analyzed, representing approximately 4% of total cases on average. Re-entry after treatment abandonment accounted for an average of 5.9% of cases between 2015 and 2020. However, a noticeable increase in this category was observed from 2021 onward, reaching 10.03% in 2021 and 13.38% in 2022.

Treatment outcomes

**Figure 3:**
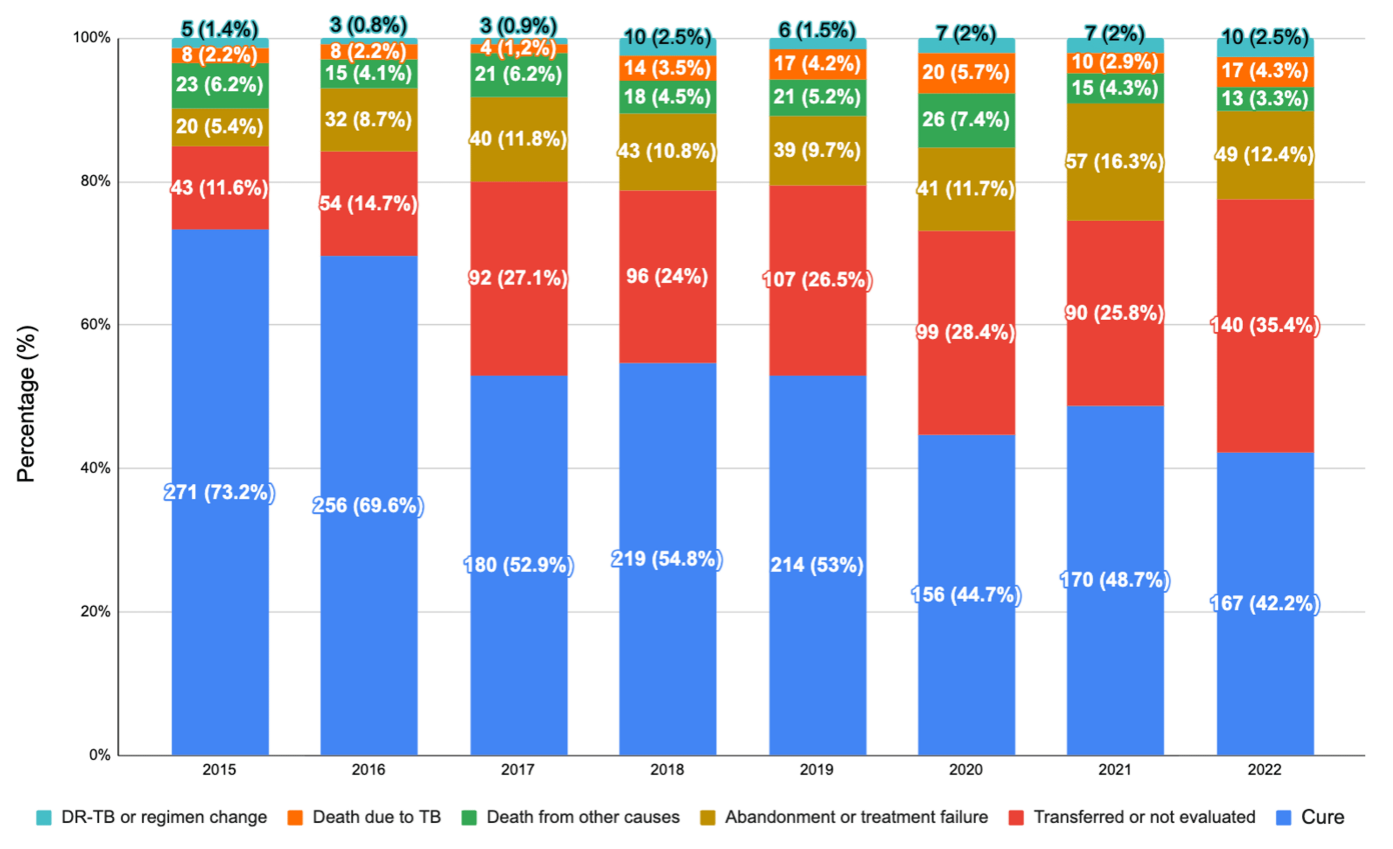
Treatment outcomes of tuberculosis cases in the Federal District, Brazil, from 2015 to 2022. Values are presented as absolute numbers (n) and percentages (%). Cure remained the most frequent treatment outcome throughout the study period; however, a progressive decline was observed over time. Cure accounted for 73.2% of cases in 2015 and 69.6% in 2016, decreasing to 52.9% in 2017 and 54.8% in 2018. During the COVID-19 pandemic period, cure rates declined further, with 156 cases (44.7%) in 2020, 170 cases (48.7%) in 2021, and 167 cases (42.2%) in 2022. The proportion of cases classified as transferred or not evaluated increased substantially over the years, rising from 43 cases (11.6%) in 2015 to 140 cases (35.4%) in 2022. Treatment abandonment or treatment failure increased from 20 cases (5.4%) in 2015 to a peak of 57 cases (16.3%) in 2021. Deaths due to tuberculosis ranged from 2.2% to 5.7%, with the highest proportion observed in 2020. Deaths from other causes accounted for 4.1% to 7.4% of cases across the study period. Cases involving drug-resistant tuberculosis or requiring a change in therapeutic regimen remained infrequent, representing approximately 1-2.5% of cases annually.

Treatment outcomes of TB cases in the Federal District were analyzed according to the situation at case closure, including cure, treatment abandonment or treatment failure, death due to TB, death from other causes, transferred or not evaluated, and drug-resistant TB or change of therapeutic regimen (Figure 3).

Throughout the study period, cure remained the most frequent treatment outcome; however, a progressive decline in cure rates was observed over time. In 2015 and 2016, cure accounted for 73.2% and 69.6% of cases, respectively. From 2017 onward, cure proportions decreased, reaching 52.9% in 2017 and 54.8% in 2018, followed by a further reduction during the COVID-19 pandemic period, with cure rates of 44.7% in 2020, 48.7% in 2021, and 42.2% in 2022. This decline should be interpreted with caution, as it may reflect disruptions in patient follow-up, continuity of care, and reporting processes during the pandemic period rather than changes in the effectiveness of TB treatment itself.

The proportion of cases classified as transferred and not evaluated increased substantially over the study period, representing 11.6% of cases in 2015 and rising to 35.4% in 2022. This increase likely reflects operational and reporting challenges, including service reorganization and limitations in case closure documentation, particularly during the COVID-19 pandemic.

Treatment abandonment or treatment failure also showed an upward trend, increasing from 5.4% in 2015 to 11.8% in 2017, with the highest proportion observed in 2021 (16.3%), followed by a slight reduction in 2022 (12.4%).

Deaths attributed to TB remained relatively stable but increased during the pandemic period, ranging from 2.2% in 2015 and 2016 to a peak of 5.7% in 2020. Deaths from other causes varied between 4.1% and 7.4% across the study period, with the highest proportion observed in 2020.

Cases involving drug-resistant TB or requiring a change in therapeutic regimen were consistently infrequent, representing approximately 1-2.5% of cases annually.

Directly observed treatment (DOT)

Coverage of DOT among TB cases in the Federal District was evaluated over the study period (Table 2). In 2015, 47.3% of patients were followed under DOT. A marked reduction in DOT coverage was observed in subsequent years, particularly from 2020 onward, reflecting the impact of pandemic-related restrictions on health service organization and patient follow-up. In 2020, DOT coverage decreased to 29.23%, remained low in 2021 (31.81%), and increased slightly in 2022 (35.10%), although it was still below pre-pandemic levels.

**Table 2:** Coverage of directly observed treatment (DOT) among tuberculosis cases in the Federal District, Brazil, from 2015 to 2022. The table summarizes the coverage of directly observed treatment (DOT) among tuberculosis cases diagnosed in the Federal District between 2015 and 2022. DOT status is categorized as “Yes,” “No,” or “Unknown/blank,” according to information recorded in the SINAN database. Results are presented as absolute numbers (n) and percentages (%), calculated based on the total number of tuberculosis cases reported for each year. Values are presented as absolute numbers (n) and percentages (%).

Year of diagnosis	Unknown/blank, n (%)	Yes, n (%)	No, n (%)	Total, n (%)
2015	88 (23.8)	175 (47.3)	107 (28.9)	370 (100)
2016	86 (23.4)	199 (54.1)	83 (22.6)	368 (100)
2017	101 (29.7)	143 (42.1)	96 (28.2)	340 (100)
2018	156 (39.0)	161 (40.3)	83 (20.8)	400 (100)
2019	188 (46.5)	134 (33.2)	82 (20.3)	404 (100)
2020	182 (52.1)	102 (29.2)	65 (18.6)	349 (100)
2021	160 (45.8)	111 (31.8)	78 (22.4)	349 (100)
2022	169 (42.7)	139 (35.1)	88 (22.2)	396 (100)

Between 2020 and 2022, a substantial proportion of cases lacked recorded information regarding DOT follow-up. On average, 46.9% of TB cases during this period had no documentation on the presence or absence of DOT and were classified as unknown or missing. This high proportion of missing or unknown DOT information represents an important limitation in surveillance data quality, particularly during the COVID-19 pandemic, when routine follow-up activities and data recording were likely compromised.

## Discussion

The findings of this study indicate that TB remains a significant public health challenge in the Federal District, even in a region with comparatively lower incidence rates. The analysis revealed that the COVID-19 pandemic had a substantial impact on TB surveillance, diagnosis, and continuity of care, reversing trends observed in the pre-pandemic period [7].

The marked reduction in TB case notifications observed in 2020 and 2021 is consistent with national surveillance reports and international evidence [7-9]. In line with global observations, this decline should be interpreted with caution, as it likely reflects under-reporting and reduced access to health services caused by the COVID-19 pandemic rather than a true reduction in disease burden [4,8]. Such trends are primarily attributed to disruptions in health services, reallocation of health professionals, and population fear of exposure to SARS-CoV-2 in health care settings, all of which created significant barriers to diagnosis and reporting during the crisis [4,9]. These factors represent a plausible explanation for the observed trends, suggesting that the reduction in notifications is tied to health system barriers rather than a change in the underlying disease dynamics.

In addition to reduced case detection, the study demonstrated a decline in treatment success indicators, particularly cure rates, accompanied by increased treatment abandonment and a substantial rise in cases classified as transferred or not evaluated from 2020 onward. This finding is further supported by the observed increase in cases classified as re-entry after treatment abandonment from 2021 onward, indicating difficulties in maintaining continuity of care during and after the pandemic period. Treatment interruption is a critical concern in TB control, as it contributes to ongoing transmission, increased risk of relapse, development of drug resistance, and higher mortality [10-13]. Similar patterns have been reported in other Brazilian regions, reinforcing the vulnerability of TB control programs during periods of health system crisis [6,14].

DOT is a cornerstone strategy for improving treatment adherence and ensuring successful outcomes. The reduction in DOT coverage observed during the pandemic period, along with the high proportion of missing information in surveillance records, highlights operational weaknesses in service organization and data quality [2,15]. The substantial proportion of cases without recorded information on DOT follow-up during this period further suggests limitations in routine data reporting under conditions of health system strain. These challenges emphasize the need for strengthening monitoring strategies and improving the completeness and reliability of surveillance data [15].

The predominance of TB among men and individuals aged 20 to 49 years observed in the Federal District aligns with findings from previous studies and reflects the disproportionate impact of the disease on the economically active population [11]. This distribution is further supported by population estimates from the Brazilian Institute of Geography and Statistics, which indicate that this age group represents the largest share of the local population [16]. These findings highlight the influence of social and structural determinants, such as working conditions, income, housing, and access to health services, on TB vulnerability and treatment outcomes [3,14].

Taken together, these findings highlight that the pandemic not only disrupted TB services but also exposed pre-existing structural weaknesses in surveillance, care coordination, and social protection mechanisms. Addressing these challenges requires integrated strategies that strengthen primary health care, improve data quality, and prioritize patient-centered approaches in TB control.

This study has limitations inherent to the use of secondary data from SINAN, including underreporting, missing information, and potential inaccuracies in records. The high proportion of incomplete data, particularly regarding DOT and treatment outcomes during the pandemic period, may have influenced the observed results. Incomplete reporting was especially frequent during the pandemic years, which may have affected the precision of some estimates presented in this study. Additionally, the descriptive design does not allow for causal inferences. Despite these limitations, the use of official surveillance data provides a comprehensive overview of TB trends in the Federal District over time.

## Conclusions

TB remains a relevant public health problem in Brazil, and although the Federal District presents lower incidence rates compared with national averages, significant challenges in disease control persist. The COVID-19 pandemic profoundly affected TB surveillance and care, resulting in reduced case detection, decreased cure rates, lower coverage of directly observed treatment, and increased treatment abandonment.

Post-pandemic recovery efforts must prioritize strengthening surveillance systems, restoring access to diagnostic and treatment services, improving data quality, and implementing patient-centered strategies to promote treatment adherence. These actions are essential to improve surveillance indicators, increase treatment completion, and ensure continuity of care, mitigate the long-term impacts of service disruptions, and support the achievement of national and global TB elimination targets.
